# The Improbable Transmission of *Trypanosoma cruzi* to Human: The Missing Link in the Dynamics and Control of Chagas Disease

**DOI:** 10.1371/journal.pntd.0002505

**Published:** 2013-11-07

**Authors:** Pierre Nouvellet, Eric Dumonteil, Sébastien Gourbière

**Affiliations:** 1 Laboratorio de Parasitología, Centro de Investigaciones Regionales “Dr. Hideyo Noguchi”, Universidad Autónoma de Yucatán, Mérida, Yucatán, Mexico; 2 EA 4218 UPVD ‘Institut de Modélisation et d'Analyses en Géo-Environnement et Santé’, Université de Perpignan Via Domitia, Perpignan, France; 3 Medical Research Council Centre for Outbreak Analysis and Modelling, Department of Infectious Disease Epidemiology, Imperial College London, London, United Kingdom; 4 Department of Tropical Medicine, School of Public Health and Tropical Medicine, Tulane University, New Orleans, Louisiana, United States of America; 5 Centre for the Study of Evolution, School of Life Sciences, University of Sussex, Brighton, United Kingdom; RTI International, United States of America

## Abstract

Chagas disease has a major impact on human health in Latin America and is becoming of global concern due to international migrations. *Trypanosoma cruzi*, the etiological agent of the disease, is one of the rare human parasites transmitted by the feces of its vector, as it is unable to reach the salivary gland of the insect. This stercorarian transmission is notoriously poorly understood, despite its crucial role in the ecology and evolution of the pathogen and the disease. The objective of this study was to quantify the probability of *T. cruzi* vectorial transmission to humans, and to use such an estimate to predict human prevalence from entomological data. We developed several models of *T. cruzi* transmission to estimate the probability of transmission from vector to host. Using datasets from the literature, we estimated the probability of transmission per contact with an infected triatomine to be 5.8×10^−4^ (95%CI: [2.6 ; 11.0]×10^−4^). This estimate was consistent across triatomine species, robust to variations in other parameters, and corresponded to 900–4,000 contacts per case. Our models subsequently allowed predicting human prevalence from vector abundance and infection rate in 7/10 independent datasets covering various triatomine species and epidemiological situations. This low probability of *T. cruzi* transmission reflected well the complex and unlikely mechanism of transmission via insect feces, and allowed predicting human prevalence from basic entomological data. Although a proof of principle study would now be valuable to validate our models' predictive ability in an even broader range of entomological and ecological settings, our quantitative estimate could allow switching the evaluation of disease risk and vector control program from purely entomological indexes to parasitological measures, as commonly done for other major vector borne diseases. This might lead to different quantitative perspectives as these indexes are well known not to be proportional one to another.

## Author Summary

Chagas disease is a parasitic disease affecting about 10 million people, often living in poor conditions, and the disease contributes to impede their development. As several other infectious diseases (malaria, dengue or sleeping sickness), it is transmitted by blood-feeding insect vectors. While most other human pathogens are directly injected with the vector's saliva, Chagas disease parasite is transmitted through the insect's feces that are deposited on the skin during bloodmeals, which seems to be a very inefficient process. The probability of such transmission to human has thus been very hard to estimate, although it is crucial to predict where people are at risk and design effective control strategies. Using mathematical models integrating data on vectors and humans collected across Latin America, we estimated that for several vector species transmission occurs in 1 over 900–4000 contacts with infected insects. We further showed that our estimate allows prediction of human infection rates in various ecological conditions. These models will provide health policy makers with improved indexes to better prioritize/evaluate of the outcomes of vector control programs.

## Introduction

Vector-borne diseases represent one of the biggest challenges to current and future human wellbeing. They have severe impacts on many tropical and subtropical countries, where they are responsible for ∼10% of human deaths and contribute to impoverishment by imposing an annual burden of >50 millions of DALYs [1]. They also are an emerging threat for more developed countries as climate change and increasing international exchanges modify the geographic distributions of vectors and pathogens [2].

Vectorial transmission is traditionally thought to critically depend on the incubation period and the survival rate of the pathogen in the vector, and on the frequency of vector feeding on humans. This is well reflected in classical measures of transmission such as the vectorial capacity, entomological inoculation rate or the basic reproductive number, which are central to empirical and theoretical literature on the ecology, evolution and control of vector-borne diseases [3], [4]. Also appearing in these standard measures is the parasite transmission efficacy from infected vectors to hosts, whose effects on vector-borne diseases has received less attention, and has frequently been assumed to be systematic, in particular for malaria transmission [5]. However, efficacy of vector transmission may become a key parameter when it takes on unusually low values, as even small variations could then have major effects on disease dynamics and the resulting prevalence in hosts [6].

The vast majority of causal agents of human vector-borne diseases, such as *Plasmodium*, *Leishmania*, dengue and other flaviviruses, are ‘salivarian’ pathogens. After entering the vector during a blood meal, the pathogens multiply inside the gut or haemolymph before spreading to the salivary glands to be directly injected to a human or a reservoir host during a subsequent blood meal. The probability of transmission from vector to a given host species is a complex process that depends on the size of the inoculate and on the within-host dynamics following inoculation. For some salivarian pathogens, the number of pathogens injected at the biting site can be measured [7], as well as the subsequent dynamics of the host-pathogen interactions [8]. Quantitative assessments of the overall resulting probability of transmission based on experimental infections gave values of 0.5 to ∼1 per bite for *Plasmodium* spp. and dengue virus, 0.3–0.6 for African trypanosome, 0.2–0.4 for *Leishmania* spp., and as low as 0.01–0.04 for the Japanese encephalitis virus [6], [9].

There also are pathogens for which this probability of transmission can be much lower as they are unable to reach the salivary glands of the vector. The so-called ‘stercorarian’ transmission, sometimes considered as the ancestor of salivarian transmission [10], occurs through the contact of vector's feces and the biting wound (or a mucosa). Successful transmission requires an extraordinary combination of somewhat unlikely events. An infected vector has to defecate sufficiently close to the biting site whilst or shortly after feeding, the infected feces must be brought to the bite wound by the host by scratching, and the pathogen then has to cross the skin of the host to initiate infection [11].

*Trypanosoma cruzi* is one of the rare parasites that has managed to establish an endemic human infection through this transmission route, and despite its presumably low probability of transmission from vector to human, it has become a major public health problem. It is indeed the etiological agent of American trypanosomiasis, also called Chagas disease, a widely distributed neglected tropical disease in Latin America, with an estimated 8–9 million infected persons and another 25–90 million at risk of infection [12]. Although maternal and oral transmissions have been documented [13], vectorial transmission remains the main cause of human infection. This protozoan kinetoplastid parasite is transmitted by a large diversity of hematophagous bugs of the Reduviidae family to multiple species of sylvatic and domestic mammalian hosts, and at least 20 species of triatomines are involved in transmission to humans [14]. Nonetheless, parasite transmission through these highly diverse vector and host communities remains poorly understood, mostly because the probability of stercorarian transmission can hardly be estimated from experimental infection due to the complexity and rareness of the process. Estimates thus rely on indirect approaches based on a combination of entomological and epidemiological studies at fine temporal and spatial scales. Given the difficulty to collect such integrative datasets, there are currently only three estimates of the probability of stercorarian transmission of *T. cruzi* to its hosts. This probability was found to be 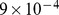 per contact with an infected vector for transmission to human [11], 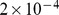 to guinea pigs, a typical domestic host in many Latin American regions [15], and 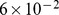 to opossums, the likely ancestral mammalian host of *T. cruzi* [16]. Although these point estimates seem rather consistently low, their usefulness remains limited as there is no information on their confidence intervals, and no sensitivity analysis to uncertainties in the entomological and epidemiological raw data has been performed. A significant benefit of gaining a robust estimate of the probability of stercorarian transmission would be to establish a clear link between basic entomological data and the prevalence of *T. cruzi* infection in humans. Potentially, this could allow evaluating disease risk and vector control program in terms of parasitological rather than entomological indexes, as commonly done for other human vector borne diseases such as malaria [4] or dengue [17].

In this contribution we thus aimed at (i) providing a robust quantitative estimate of the probability of stercorarian transmission of *T. cruzi* to humans based on information available in the literature, and at (ii) determining if this estimate allows predicting the prevalence of the infection from basic entomological data.

## Materials and Methods

### 1. General overview

Our estimate of the probability of stercorarian transmission of *T. cruzi* to humans was derived from an indirect approach relying on a standard mathematical relationship which links the number of healthy hosts to become infected, i.e. the incidence of *T. cruzi* infection, to the average number of potentially infective contacts per individual host, and the probability of pathogen transmission per potentially infective contacts [4]. Given such a relationship, field measurements of the first two quantities allow estimating indirectly the probability of transmission per contact from infected vector to host.

We expanded this standard modelling of transmission to derive the expected distribution of the number of susceptible humans acquiring the parasite in order to obtain a maximum likelihood estimate of the probability of stercorarian transmission. We first used this framework to re-analyze the data from [11], which provides entomological (vector abundance, infection rate, feeding frequency and the proportion of blood meals on humans) and epidemiological (incidence of infection in humans) data at the household scale. Next, we adjusted our model to estimate the probability of transmission to humans (i) when entomological and/or epidemiological data are available at the village rather than at the household scale, and (ii) when epidemiological data consist of human prevalence rather than incidence. These adjustments allowed estimating the probability of stercorarian transmission from four additional datasets. We then performed sensitivity analysis of each estimate to the uncertainties in the measurement of the entomological variables.

Finally, we aimed at testing whether our modelling framework and estimates would allow predicting the prevalence of *T. cruzi* infection from limited entomological data. We thus predicted the prevalence of infection in different human populations representing nine different epidemiological settings, based on vector abundance and its *T. cruzi* infection rate combined with our estimate of the stercorarian transmission probability, and compared these predictions to the observed human prevalence.

### 2. Modelling of vector to human transmission

Vectorial transmission of *T. cruzi* to humans typically takes place inside the domestic habitat, and an accurate description of transmission thus needs to focus at this scale. Considering a household 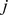
(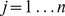) with 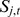 susceptible individuals, the probability of observing 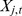 new cases during a finite period of time 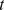, follows a binomial law characterised by the probability 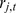 for a susceptible human from that house to become infected during that period:
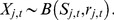
(1)The probability 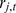 can be linked to the per contact transmission probability of parasites from an infected vector to a susceptible human, 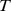, and the per human number of potentially infectious contacts with vectors during a single time unit, 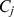. Formally, and as in [11], we have for the household ‘
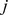
’:
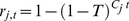
(2a)which, as *T* is typically small for *T. cruzi*, can be approximated by the catalytic model [18], [19];
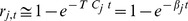
(2b)where 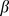 stands for the ‘force of infection’ [19] at the household scale.

The number of potentially infectious contacts per human per time unit can, in turn, be derived from the number of vectors present, 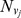; the proportion of infectious vectors, 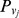; the biting rate of vectors per time unit, 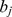; the feeding rate on humans, 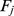; and finally the total number of individuals in the household, 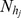:
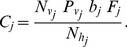
(3)Combining equations 2 and 3 leads to a non-linear relationship between the abundance of vectors and the incidence in humans.

### 3. Estimating the probability of transmission of *Trypanosoma cruzi* to humans

When all parameters appearing in equations 1–3 are known from field measurements at the household scale, the probability *T* of transmission can be estimated together with a confidence interval using a standard maximum likelihood approach [20]. Using Eqs. 1–3 the log likelihood function to be maximized can be defined as:
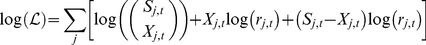
(4)where 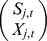 stands for the binomial coefficient. An interval estimate of *T* is then obtained drawing a maximum likelihood profile as a function of *T* [20].

However, in most cases, entomological and/or epidemiological data are available at the village rather than at the household scale (see Table 1). Still, a point estimate of *T* can be derived from Eqs. 1–3, but this requires further assumptions and calculations. Primarily, we have to assume that the parameters appearing in Eq. 3 have the same value across houses, leading to a common number of potentially infectious contacts per human (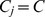). Under such an assumption, the probability to become infected is the same in each household (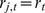), and the distribution of incidence for the entire village can be modelled by a unique Binomial 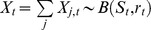, where 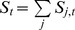 stands for the total number of susceptible individuals in the village. The expectation of this law provides an estimate of 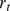 as the ratio between numbers of newly infected and susceptible individuals summed over all houses: 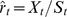. From Eq. 2b, one can derive a simple point estimate of *T*:
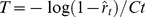
(5)In addition, epidemiological studies most frequently report human prevalence rather than incidence. Here again, one can use Eqs. 1–3 to derive a point estimate of *T* under simple additional assumptions. Prevalence typically results from a dynamical equilibrium between the rate at which individuals become infected and the rate at which they die. Assuming that both the force of infection, 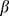, and the human death rate, 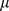, are constant over time, the prevalence observed in the whole population, 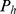, can be used to estimate the force of infection; 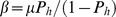 (see Appendix S1 for details). Using equation 2b, one can then propose another simple point estimate of *T*;
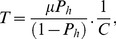
(6)Prevalence data are sometimes provided for children under a given age *A*, 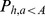. The force of infection 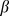 can still be derived under similar assumptions, although it has to be evaluated numerically from the following equation (see Appendix S1 for calculations):
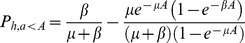
(7)From Eq. 2b one can again derive a point estimate of *T* from the estimated value of 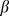 since
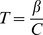
(8)We again point out that both equations 6 or 7 are consistent with other known non-linear relationships between the abundance of vectors and the prevalence of infection in humans. Box 1 provides a guideline to incorporate the entomological and epidemiological knowledge into the modelling proposed in this contribution. Together with Appendices S1 and S2, it also summarizes the assumptions of the models and the potential limitation of this integrative approach.

**Table 1:** Entomological and epidemiological datasets used to estimate the probability of *T. cruzi* transmission from vector to human (*T*) per contact with infected vector.

Dataset	Species and location	Corrected vector density ( 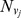 )	Vector infection rate ( 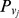 )	Biting rate ( 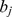 )	Feeding rate on humans ( 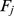 )	Human per house ( 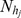 )	PIC (per year, 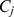 )	Incidence (yearly)	Source
**1**	*T. infestans*, Gran Chaco, Argentina	Per houseM1 ^,^ a: 287±47	Per house 37%±5	From^c^: 0.4	Per house: 46%±4	Per house: 7±0.6	340±72	Directly in children^e^: 20.26%±8.86	[11]
**2**	*T. infestans*, South of Gran Chaco, Argentina	Per houseM1 ^,^ a: 140±32	Per house: 10%±3	Per house^c^: 0.3±0.04	Per house: 42%±6	Per house: 5±0.6	164±77	From prevalence in children^d^ (Eq. 7–8, 2b): 2.54%	[24]
**3**	*T. infestans*, Gran Chaco, Argentina	Per houseM1 ^,^ a: 80±15	Per house: 40%±5	Average from^c^: 0.4	Per house: 40%±5	Per house: 5±0.5	248±56	Directly in children^f^ (Eq. 5, 2b): 4.21%	[25], [26]
**4**	*T. longipennis* and *barberi*, Jalisco, Mexico	Average per houseM1: 45	Average for the village: 46%	Average from^c^: 0.2	Average for the village: 1%	Average per house in the village: 4	4	From prevalence (Eq. 6, 2b): 0.08%	[27], [28]
**5**	*T. dimidiata*, Yucatan, Mexico	Average per houseM2 ^,^ b: Teya: 40, Sudzal: 20, Merida: 1	Average for the village: Teya: 14%, Sudzal: 27%, Merida:48%	Average from^c^: 0.2	Average for the village: Teya: 6%, Sudzal: 6%, Merida:26%	Average per house in the village: 4	Teya: 7, Sudzal: 7, Merida: 3	From prevalence (Eq. 6, 2b): Teya: 0.26% , Sudzal: 0.68% , Merida: 0.26%	[29], [30],[31]

Data are presented as mean ± SE (when available in the original study). Entomological data were combined to estimate the number of potentially infectious contact per person per year (PIC).

^M1^ and ^M2^ refer to the triatomine collection methods and the corresponding correction factors defined in the main text.

a Vector densities were further corrected for seasonality using monthly variations in infestation of *T. infestans* [32] or

b for the seasonal infestation pattern of *T. dimidiata* [21].

c The biting rate was estimated and corrected for seasonal variations according to [33]. Incidence was either measured directly or derived from prevalence using data on children or on all age-categories at the house or village scale.

d children <15 years old,

e incidence after 2 years of exposure at the household scale,

f incidence after 3 years of exposure at the village scale.

**Box 1: Models assumptions, applicability and associated caveats**

Case 1. When entomological and epidemiological information are available at the household scale, a likelihood estimation is possible. These data include the abundance of vectors (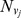), the prevalence of infection in vectors (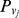), the vector biting rate (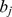), the feeding rate on humans (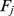) and the number of individuals in the household (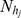). The analysis relies on equations 1–4 assuming that:

- All parameters used in the calculation of the risk per household, 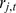, are determined without errors and are constant during the period over which the incidence is measured.
- The probability of transmission, *T*, is a constant, in particular human of different ages have the same probability of acquiring the parasite and we assumed no loss of infection because of successful treatment.
- The vector's feeding rate on human does not vary with human age.

Case 2. When the above information is not available at the household scale, we used aggregated data and equation 5 which assumes, in addition to all assumptions described above, that:

- The number of potentially infectious contacts per human per time unit is constant across houses; 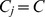, and estimated from the aggregated values of each of the entomological and epidemiological parameters. This assumption is most likely wrong and would induce a bias when vector/human contacts are spatially heterogeneous. However, we estimated that this bias would not exceed a 20% underestimation in the worst case scenario (i.e. highly heterogeneous distribution of vectors, see Appendix S2 for details).

Case 3. When information on human incidence is not available, we use a catalytic model to infer incidence values from either prevalence in the total population (equation 6) or prevalence per age class (equation 7). Using this approach we assumed, in addition to all assumptions above, that:

- The human mortality is constant across ages.
- The entomological observations made at time *t* must reflect the conditions in which the prevalence was measured. For instance, if using prevalence data of human under 20 years old, then entomological data are assumed to have been constant during the 20 previous years.

This last assumption is critical, and to limit potential bias, prevalence in younger groups should be used to best reflect the contemporary entomologic conditions (see table 2).

The sensitivity analyses relax the group of assumptions 1), and while informative, we stress that we would need better data not better models to improve our estimate of the probability of transmission of *T. cruzi* to human.

**Table 2:** Entomological datasets used to predict human prevalence.

Dataset	Species and location	Corrected vector density 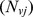	Vector infection rate 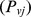	Biting rate (*b_j_*)	Feeding rate on humans (*F_j_*)^a^	Human per house 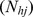	PIC (per year,*C_j_*)	Source
6	*T. brasiliensis*, *T. pseudomaculata*, Brazil	1.0 (D); 9.6 (P)^M1^	12% (D); 16% (P)	0.2	26% (D); 1% (P)	4	1.3	[34], [35]
7	*T. pallidipennis*, *T. longipennis*, Mexico	4.2 (D); 2.6 (P)^M3^	26%	0.2	26% (D); 1% (P)	4	5.2	[36], [37]
8	*T. infestans*, Peru	17.8 (D); 58.2 (P)^M1^	15% (D); 10% (P)	0.4	40% (D); 1% (P)	6	13.0	[38], [39]
9	*T. barberi*, *T. mexicana*, *T. dimidiata*, Mexico	5.4 (D)^M2^	7% (D)	0.2	26% (D)	4	1.7	[40]
10	*T. dimidiate*, Costa Rica	1.8 (D); 2.4 (P)^M1^	35% (D); 30% (P)	0.2	26% (D); 1% (P)	4	8.1	[41]
11	*R. prolixus*, *P. geniculatus*, Venezuela	10.4 (D)^M3^	17% (D)	0.2	Observed: 0.583	4	18.6	[42]
12	*T. infestans*, Paraguay	43.5 (D)^M3^	20% (D)	0.4	40% (D)	6	46.3	[43]
13	*R. prolixus*, *T. maculata*, Venezuela	8.6 (D)^M2^	1% (D)	0.2	26% (D)	4	0.4	[44]
14	*T. infestans*, Bolivia	SZ: 16.6 (D), NZ: 8.3 (D)^M2^	SZ: 79% (D), NZ: 37% (D)	0.4	40% (D)	7	SZ: 56.4, NZ: 13.4	[45]

Basic entomological data (vector density and infection rate) and the estimate of the probability of transmission were used to predict human prevalence. Vector densities (average per house) were corrected according to the collection methods used (M1, M2 and M3) as defined in the main text. Density and infection rates were available for domestic (D) or peridomestic (P) habitats. Mean infection rates are given for the villages. The biting rate was set up to 0.2, i.e. the average rate estimated by [33].

a The proportion of blood-meals on humans was selected according to the habitat of the bugs. For *T. infestans*, this proportion was set to 0.4 in the domestic habitat [11], [24] and to 0.01 in the peridomicile [27]. For other species, this proportion was set to 0.26 [46] and 0.01 in the domestic and peridomestic habitat, respectively. SZ, NZ: Respectively south and North zone of Cochabamba, Bolivia [45].

### 4. Sensitivity analysis of the estimate of the transmission probability

The assessment of the probability of transmission *T* using the indirect approaches described above relies on the estimates of the various quantities appearing in equations 1–3. Since they all are subject to estimation uncertainty (Table 1), we performed a sensitivity analysis to such uncertainties in 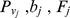 and 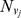 by determining the distribution of *T* estimates that resulted from variations in the raw data.

The distributions of plausible estimates were obtained by randomly sampling 1000 values of 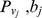 and 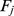 in independent zero-truncated normal distributions with mean and standard deviation estimated from the data (Table 1). When no information on the variability in measurements was reported by the authors, we used the standard deviation calculated from dataset 2, as this dataset provides the most comprehensive information about these three parameters. When species-specific information was missing, species-aggregated estimates were used. Since the average values of these parameters were typically larger than 5% (Table 1), sampling from an exact zero truncated binomial distribution would not change the results of the sensitivity analysis.

The sensitivity analysis to the density of vectors, 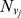, was performed in a slightly different manner. Since bug collection tends to underestimate vector densities, a correction factor is usually applied to estimate actual vector densities according to the efficacy of the particular collection method. Two different methods were used in these studies; M1 - timed-manual collection with insecticide spraying or aerosol to kill or dislodge triatomines (datasets 1–4), and M2 - passive surveillance by inhabitants or community participation (dataset 5). The correction factor of M1 and M2 have been previously estimated to be around 2.5 [11] and 10 [21], respectively. The sensitivity analysis of the estimate of *T* to variations in *N_vj_* was performed by varying the correction factor according to a uniform distribution within a range defined as its value ±1.5. This range was chosen as the maximum possible range to guarantee that samples collected with M1 reflected at least the density of bugs actually found (i.e. for the lowest correction factor to be 2.5-1.5 = 1), while keeping the mean of the correction factor equals to the value of 2.5 that was inferred from field data [11]. For consistency the same range was applied to other collection methods (see below for the definition of a last method - M3 - used for datasets that allowed evaluation of the model predictive ability). Accordingly, the range tested for the different methods (M1 : 1–4, M3 : 4.5–7.5, M2: 8.5–11.5) did not overlap, which is consistent with the general understanding that M1 is more efficient than M2, while M3 has an intermediate efficacy.

### 5. Predicting human prevalence

We then explored the usefulness of our models and our best estimate of the probability of transmission *T* (i.e. from dataset 1, see results) to predict the prevalence of infection in humans. From the literature, we selected nine additional studies (Table 2) reporting data on triatomine density and infection rates, as well as human prevalence of infection, so that predictions of human prevalence could be derived and compared with the observed values.

As for the sensitivity analyses above, uncertainties in the raw data were taken into account for the predictions of prevalence of infection in humans. We first constructed a distribution of the number of potentially infectious contacts per human (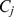) accounting for the variability in 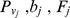 and 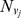, according to the same zero-truncated normal and uniform distributions as described for the estimates of *T*. The standard deviations of the zero-truncated normal distributions were again estimated from the data whenever possible, or we used the standard deviation calculated from the most comprehensive dataset (dataset 2). The range of the uniform distribution was determined according to the insect collection method as described above. However, in 3 case studies (datasets 6, 11 and 12), bugs were collected using a third method; M3 - a timed-manual collection by trained personnel without insecticide spraying. We considered this method to have an intermediate efficacy, and we thus set the correction factor to 6 and varied its value within a range set to its value ±1.5.

We then determined the expected distribution of the number of infected individuals by sampling into the binomial distribution given by equation 1, with probabilities 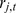 calculated from *T* and the distribution of 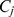, and with the number of susceptible humans 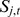 given by the number of individuals that were tested for Chagas disease in the prevalence studies. Since the expected number of infected individuals could be low, we applied a continuous form of the binomial distribution [22] and sampled it using the ‘acceptance-rejection’ methods [23]. This allowed determining a 95% confidence interval for the expected prevalence, and calculating a p-value as the probability for the observed prevalence to belong to the distribution of predicted prevalence.

### 6. Entomo-epidemiological datasets

#### Estimate of the probability of transmission (Datasets 1–5)

Estimating the probability *T* of transmission requires detailed entomological and epidemiological data, which are only available from long-term field studies on specific biological systems. We thus re-analyzed the data presented by [11], and extensively reviewed the literature in the field of Chagas disease to identify additional systems which have been consistently investigated in the field over the last 10–15 years so that all entomological and epidemiological data required to get estimates of the probability of transmission were available (Table 1, [24]–[33]). These studies involve four vector species (*Triatoma infestans*, *T. longipennis*, *T. barberi* and *T. dimidiata*) from five locations in Argentina and Mexico. They described various entomological settings with corrected vector density varying from less than 10 to almost 300 triatomines per house, vector infection rate ranging from 10% to 58%, and vector feeding frequency on human of 1% to 46%. Overall, those entomological and epidemiological heterogeneities combined to produce substantial variations in the number of potentially infectious contacts that ranged from 3 to 340 contacts per human per year which, as expected, correlated with annual incidence of infection that ranged from about 0.1% to 20% (Table 1).

The first dataset provided all the entomological and epidemiological information at the house scale, allowing to calculate a log likelihood function (Eq. 4) and to derive an estimate and confidence interval for the probability of transmission *T*. In all the remaining datasets, some entomological (datasets 4–5) and/or epidemiological (datasets 2–5) data were only available at the village scale, so that only point estimates could be derived. When *T. cruzi* incidence was given in the original study (dataset 3), the estimate was calculated using Eq. 5, while when prevalence was measured in adult (datasets 4–5) or in children (2), the point estimate of *T* was calculated from Eq. 6 and 7–8, respectively.

#### Predicting human prevalence (Datasets 6–14)

To evaluate whether our estimates of the probability of transmission *T* would allow predicting the prevalence of infection in humans from limited entomological knowledge, we selected nine independent case studies reporting data on the average triatomine density and infection rate, and on human prevalence of infection (Table 2, [34]–[46]). Again, these data were obtained by careful screening of the literature. Estimates of the biting frequency and proportion of bites on human were not available in those studies, and we thus used values from dataset 1 to 5 according to the species of interest (i.e. *T. infestans* .vs. others) and the habitat of the vector (i.e. domicile .vs. peridomicile). These new datasets covered 7 countries from South and Central America, and 11 vector species including the 3 previous species, *T. infestans*, *T. barberi* and *T. longipennis*, as well as *T. brasiliensis*, *T pseudomaculata*, *T maculata*, *T. mexicana*, *T. dimidiata*, *T. pallidipennis*, *T. longipennis*, *Rhodnius prolixus* and *Panstrongylus geniculatus*. Vector densities varied between 1 and 46 inside the house, and between 2 and 58 in the peridomiciles. This, combined with important variation in vector infection rates ranging from 1 to 79%, led to a number of potentially infectious contacts per human and per year ranging from less than 1 to 56 which, again, correlated with the observed prevalence in humans that varied from 1.6 to 29.1%.

## Results

### 1. Estimating the probability of *Trypanosoma cruzi* transmission to human

**Figure 1:**
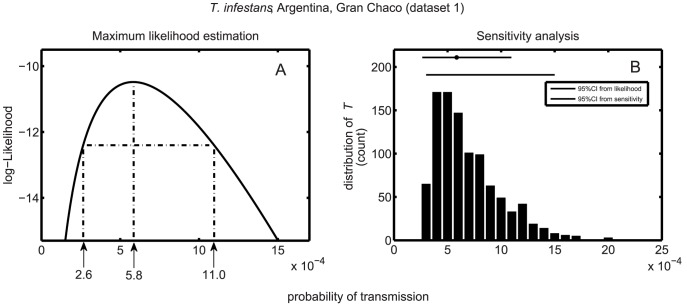
Maximum likelihood estimate of the probability of transmission of *T. cruzi*. (A) profile likelihood, maximum likelihood estimate (MLE) of the probability of transmission *T*, and its 95% maximum likelihood confidence interval (MLCI). (B) Distribution of the MLE of *T* obtained from the sensitivity analyses (1000 replications). Grey and black horizontal bars on the top of the figure represent the 95% MLCI (with the grey dot corresponding to the MLE) and the interval including 95% of the MLE estimates obtained from the sensitivity analysis.

We first determined the probability of *T. cruzi* transmission using dataset 1 which includes data collected at the scale of the household [11]. A profile likelihood was drawn as a function of the probability of transmission (Figure 1). Such profile provided a maximum likelihood estimate of 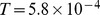 per contact with infected bugs, which was close to the original point estimate of 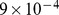 obtained with the same data. The profile likelihood also provided a confidence interval around this estimate, which was 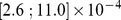 per contact, which means that on average 900–4000 contacts with infected vector are needed for a host to become infected (Figure 1). This confidence interval was confirmed by the sensitivity analysis to variations in entomological raw data (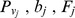 and 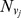). The 95% range of the sensitivity estimates was 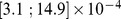 per contact and the corresponding distribution remained well within the confidence interval derived from the likelihood approach. Only 10% of values from the sensitivity analysis felt outside of the 95% likelihood confidence interval.

**Figure 2:**
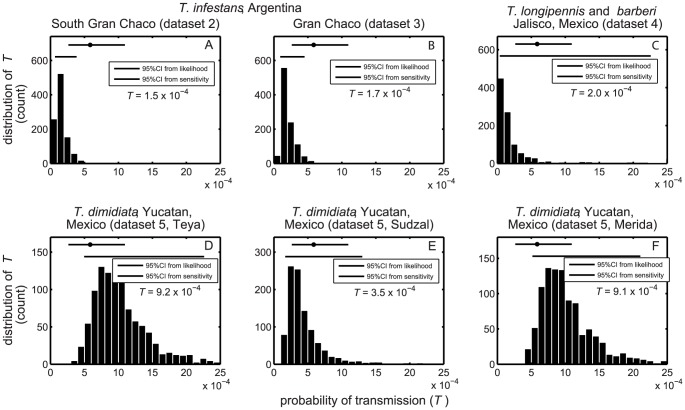
Sensitivity analyses of the probability of transmission of *T. cruzi*. Each panel gives the distribution of point estimates of T obtained from the sensitivity analyses (1000 replications). Panels A, B and C correspond to datasets 2, 3 and 4, respectively, while panels D, E and F correspond to each of the three villages included in dataset 5. Black bars represent the interval including 95% of the point estimates obtained from the sensitivity analysis. The grey dots and bars represent the maximum likelihood estimate (MLE) and 95% maximum likelihood confidence interval (MLCI) obtained from the dataset 1 for comparison (see Figure 1).

From the other four datasets, where entomological and/or epidemiological data were provided at the village scale, we derived additional point estimates of the probability of transmission of *T. cruzi* to human. These estimates varied from 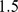 and 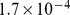 for *T. infestans* in Argentina and 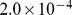 for *T. longipennis* and *T. barberi* in Mexico, to 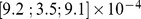 for *T. dimidiata* in Mexico (Figure 2). They all lied within or very close to the confidence interval derived above from dataset 1 indicating that there is no major difference between the maximum likelihood estimates of *T* and these four estimates. The estimate for dataset 4 can be viewed as a species-averaged probability of transmission as the data do not allow to distinguish infection from either *T. longipennis* or *T. barberi*. The sensitivity analyses to variation in the entomological raw data (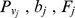 and 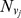) further confirmed the high consistency of those results. The distribution of estimates obtained for the other two datasets on *T. infestans* were slightly less dispersed than in the first data set (Figure 2A and B) with 95% of the values ranging in 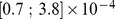 for dataset 2, and in 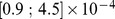 for dataset 3. The distribution obtained for *T. longipennis* and *T. barberi* (dataset 4) in Mexico were slightly broader with 95% of the estimates found within 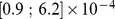 (Figure 2C). Finally the distributions obtained for *T. dimidiata* in Mexico (dataset 5), were the most variable since 95% of the values laid within 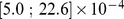, 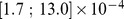 and 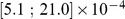 for the villages of Teya, Sudzal and the city of Merida, respectively (Figure 2D, E and F). Nevertheless, all estimates were still very consistent with the likelihood-based confidence interval. According to our analysis, including a likelihood estimation with confidence interval, point estimations and sensitivity analyses, the probability of stercorarian transmission of *T. cruzi* to human is estimated to be in the range 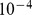 - 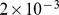 per contact.

### 2. Predicting the prevalence of *T. cruzi* infection from entomological data

We then attempted to predict human prevalence of infection based on the probability of transmission determined above and basic entomological data, using nine independent case-studies (Table 3). Those included *T. infestans* in Peru and in southern Cochabamba in Bolivia, *T. barberi*, *T. mexicana*, and *T. dimidiata* in Mexico and Costa Rica, *T. brasiliensis* and *T. pseudomaculata* in Brazil, *T. pallidipennis* and *T. longipennis* in Mexico, and *R. prolixus* and *P. geniculatus* in Venezuela.

**Table 3:** Predictions of human prevalence from basic entomological data and the probability of *T. cruzi* transmission.

Dataset	Species and location	Observed human prevalence	Predicted human prevalence [95% CI]	p-value
6	*T. brasiliensis*, *T. pseudomaculata*, Brazil	3.1% [1.7–4.6]	2.9% [1.7–4.4]	0.776
7	*T. pallidipennis*, *T. longipennis*, Mexico	2.0%^a^ [0.01–4.8], 3.0%^b^ [0.6–5.2]	2.0%^a^ [1.3–3.0], 3.5%^b^ [2.3–5.1]	0.999, 0.384^b^
8	*T. infestans*, Peru	5.3%^a^ [3.4–7.9]	6.1%^a^ [4.6–7.8]	0.335^a^
9	*T. barberi*, *T. mexicana*, *T. dimidiata*, Mexico	3.7% [1.2–6.3]	2.1% [0.1–4.3]	0.122
10	*T. dimidiate*, Costa Rica	11.7% [10.0–13.4]	10.2% [8.5–11.8]	0.068f
11	*R. prolixus*, *P. geniculatus*, Venezuela	15.5% [7.7–23.2]	25.0% [15.8–35.0]	0.050
12	*T. infestans*, Paraguay	29.1% [23.8–34.4]	36.9% [29.7–42.1]*	0.020
13	*R. prolixus*, *T. maculata*, Venezuela	1.6% [0.5–2.6]	0.7% [0.1–1.5]*	0.014
14	*T. infestans*, Bolivia	SZ: 24.9%^c^ [22.1–27.8], NZ: 18.9%^c^ [16.4–21.5]	SZ: 24.7% [22.1–27.5], NZ: 6.4% [5.2–7.6]*	SZ: 0.889, NZ:<0.001

Observed prevalences are presented together with the distribution of predicted prevalence (as described by a 95% confidence intervals) and the probability for the observed prevalence to be within the predicted distribution (*indicates a statistical difference between observation and predictions at a 95% confidence level). Prevalence of infection was measured and predicted for individuals under 15 years.

a ^a^, under 30 years.

b ^b^, or between 8 and 13 years.

c ^c^of age.

SZ, NZ: Respectively South and North zone of Cochabamba, Bolivia [45].

In seven of these cases, our model satisfactorily predicted human prevalence (Table 3), as indicated by a lack of significant differences between observed and predicted prevalence. In three cases (*T. infestans* in northern Cochabamba, Bolivia, and in Paraguay, and *T. maculata* in Venezuela) there were statistical differences between the observed and predicted prevalence. However, in the later two cases, the observed and expected prevalences of infection were of very similar magnitude and almost included in the 95% confidence interval. Accordingly, the difference between the high level of transmission by *T. infestans* in Paraguay and the weak level of transmission by *T. maculata* in Venezuela were thus properly predicted, so that our model would not lead to any lack of appreciation of a serious health issue. Only in one instance, *T. infestans* in Northern Cochabamba, Bolivia, a large discrepancy was found and could not satisfactorily be explained from available data.

We thus obtained accurate predictions of human prevalence of *T. cruzi* infection over a broad range of epidemiological conditions ranging from low to high prevalence of infection (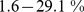), a wide geographic range (with 7 countries across Latin America), and 12 species of triatomines.

## Discussion

The lack of a quantitative estimate of the probability of *T. cruzi* transmission to human through the feces of the vector has hindered the development of approaches that integrate ecological and epidemiological information on Chagas disease. These approaches have had an impressive influence in mitigating several vector-borne diseases including malaria [47], dengue [48] or leishmaniasis [49], and would help better understand the complex features of the transmission of *T. cruzi* and compare it with other vector-borne diseases. Based on data from the literature we built here epidemiological models to derive 6 estimates of this probability of transmission, all being of the order of 10^−4^–10^−3^ per contact. This primarily illustrates the paradox of Chagas disease; despite the ‘milli-transmission’ of the parasite from vectors to humans, the disease affects millions of people across the Americas. The quantitative knowledge of its transmission probability also opens new perspectives for the study of the disease, with key implications for both parasite evolution and public health policy.

### 1. The ‘milli-transmission’ of *Trypanosoma cruzi* to humans: Possible causes

The probability of transmission of *T. cruzi* from triatomine vectors to humans was found to be very small, 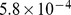 per contact with infected vector (95% CI 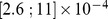), relatively consistent across the different study systems, with point estimates ranging from 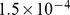 to 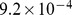, and in agreement with the only other point estimate available in the literature [11]. This narrow range of probabilities was observed in spite of marked differences in vector density, vector species (taxonomic, ecologic and behavioural differences), prevalence of infection in humans and vectors, resulting in very different epidemiological situations. Such a broad consistency was confirmed by our sensitivity analyses, which further supported that estimates are robust to changes in the entomological and epidemiological raw data used for their calculation.

These estimates of parasite transmission to human are similar to what has been calculated for guinea pigs [15], but differ substantially with the probability of stercorarian transmission to opossums that was estimated to be 10–100 times larger [17]. This suggests a reduced adaptation of *T. cruzi* to domestic hosts compared to its likely ancestral and sylvatic host, which is consistent with the much shorter period of coevolution between *T. cruzi* and humans. Indeed, estimates suggest around ∼10000 years of coevolution of *T. cruzi* with humans, compared to ∼80 millions years with the opossum [50]. Such a low probability of transmission does not mean that humans are of secondary importance or even ‘dead ends’ in term of parasite transmission, as suggested by the ∼40% prevalence of infection in humans observed in 9000-years old mummies [51] as well as in today's highly endemic areas [45]. In fact, the potential amplification and dilution effects [52] that human and other domestic hosts could have on the populations of *T. cruzi* still remain to be properly quantified (but see [53]).

A low probability of vectorial transmission of *T. cruzi* was expected, given the succession of unlikely events required to occur and the many parameters involved. However, the narrow range of probabilities was more surprising given that all of these parameters could potentially affect parasite transmission quite dramatically. This suggests that these parameters combine in an independent way to produce an almost universal efficacy of transmission of *T. cruzi* from vectors to humans. While more accurate data may allow refining our estimate of the probability of transmission of *T. cruzi* to human, potentially detecting species specificity, the residual variations in the probability of transmission are expected to have little impact on the prevalence of infection in humans. Indeed, we were able to predict rather accurately the prevalence in humans from infected vector density, the frequency of human blood meal, and a unique probability of vectorial transmission. Triatomine vectorial capacity is thus primarily dependent upon vector density and feeding frequencies on specific hosts, a conclusion which is consistent with the key influence of those parameters on the spread and persistence of the disease [6]. As vector demography has been documented for a variety of triatomine species and entomological context [21], [53], [54], [55], the emerging eco-epidemiology of Chagas disease would benefit from a substantial improvement of our knowledge on vector feeding ecology. The emergence of methods based on the use of metagenomics [56] and stable isotopes, which potentially allow identifying vector trophic networks [57], should shortly allow tackling the transmission of *T. cruzi* in the context of host communities, as it has already been done successfully for the transmission of plague [58].

The very low probabilities of transmission of *T. cruzi* from vector to vertebrate hosts raises an obvious evolutionary question: why has *T. cruzi* not evolved from a stercorarian to a salivarian mode of transmission while closely related species such as *T. rangeli* [59] and *T. brucei* [60] or *Leishmania* [61] have been able to do so? A first line of explanation could lie in a lower ‘evolvability’ of *T. cruzi* [62]. However, there is no evidence that *T. cruzi* has a lower mutation rate compared to other kinetoplastids [63], and *T. cruzi* does experience reproduction and recombination [64] as demonstrated for other related taxa [65], [66], [67]. A lower ‘evolvability’ could also result from specific features in *T. cruzi* genotypes to phenotypes mapping function, which may be evaluated by mutagenesis and artificial selection experiments [62]. A second line of explanations that could explain *T. cruzi* ineffective mode of transmission are the costs associated with the migration of the parasite across the midgut, the escape of the immune response in the haemocoel, and the invasion of the salivary glands, which all may exert selective pressure to restrict the parasite to the gut. Those costs have not been identified yet, although molecular studies are progressively unravelling the interaction between *T. cruzi* and its vector [14], [68], and insights could be gained by comparative analysis of vector-parasite interactions of the various kinetoplastids [14]. Comparisons with *T. rangeli*, a closely related and sympatric parasite that shares hosts and vectors with *T. cruzi* [69], should be especially informative as it is able to colonize the haemocoel and reach the salivary glands of its vector [59]. Finally, the selective pressure on *T. cruzi* may be too weak given its potential for direct transmission which is known to be of evolutionary and epidemiological importance in opossum [16], [70] as well as in human either because of oral or maternal transmission [13].

Our quantitative assessment of the probability of transmission of *T. cruzi* offers new opportunities to tackle these key eco-evolutionary questions, as it allows quantifying standard epidemiological measures such as R_0_ or related quantities [3], [4], [71] which have been consistently missing in the epidemiology of Chagas disease [6], [ but see 72], while they are central tenets of the study of the epidemiological dynamics of malaria [4], dengue [17] and others human and livestock vector-borne diseases [73].

### 2. Shifting the assessment of Chagas disease risk and control programs from entomological to parasitological indexes

Our study demonstrates that the quantitative estimates of the probability of *T. cruzi* transmission from vector to humans allow expressing infection risk in terms of human incidence or prevalence, rather than in terms of purely entomological indexes such as vector presence/absence [74] or abundance [75]. Importantly, the entomological data used to make those predictions are basic estimates of vector abundance and infection rates that can inferred from typical entomological collections achieved by trained personnel or even low-cost studies based on community participation [76]. Although a proof of principle study is necessary to validate the proposed models' predictive ability in an even broader range of ecological settings, this approach offers a much more affordable way than large-scale serological surveys to estimate human prevalence over geographic areas and obtain better descriptions of the global and local burden of the disease. Such prevalence/incidence data would be more straightforward and explicit to interpret at all levels of public health systems for the design of epidemiological surveillance and vector control operations. In addition, the risk expressed in incidence or prevalence would likely differ from that expressed in vector presence/absence or abundance because, according to the catalytic model [18], the relationship between these variables is non-linear and follows a cumulative exponential distribution. At low vector densities the risk of human transmission increases rapidly with vector abundance, and is thus likely to be underestimated by the sole measure of vector abundance, while at large vector densities, human incidence and prevalence reach a plateau, so that variations in vector abundance have little or no influence on the already high transmission to human.

Our model can also profoundly help the assessment of the efficacy of vector control interventions, which is traditionally measured in terms of reduction in vector abundance or vector presence (infestation index) [53], [77], [78]. Typically, current guidelines for vector control in several endemic countries aim at reducing triatomine presence below the somewhat arbitrary level of 5% of the houses of a community [79], based on the assumption that this may be sufficient to dramatically reduce or even interrupt parasite transmission to humans. The modelling developed here opens the possibility to convert a reduction of vector abundance into a variation in the actual level of parasite transmission to humans, allowing to rapidly define more rational target/threshold levels of infestation for vector control. Again, one expects that for high vector densities, very large reduction in vector populations will be needed to reduce human prevalence, while at low vector abundance, small reductions in vectors could result in significant decrease in human prevalence. Nonetheless, even small residual populations of bugs due to incomplete treatment [80], development of insecticide resistance [81] or infestation by non-domiciliated vectors [21], [55], [82], [83], [84], [85] will be sufficient to maintain an active transmission of *T. cruzi* to humans, which clearly appeals for the use of highly sensitive tools for entomological surveillance following the ‘action’ stage of control program [86].

In conclusion, we provided estimates of the probability of *T. cruzi* transmission from vector to human, which were shown to be highly consistent across vector species and epidemiological conditions. Such a new quantitative knowledge could allow expanding purely entomological indexes, which are typically calculated for triatomines, into parasitological measures (such as R_0_, the so-called force of infection and resulting incidence and prevalence), as routinely done for other human vector-borne diseases such as malaria or dengue. This offers the possibility to develop a better understanding of the ecology and evolution of one of the rare stercorarian human parasites. This also is of primary interest in public health, as parasitological measures provide a more straightforward evaluation of the disease risk and a better description of the outcomes of vector control program in terms of human infection rather than vector abundance. Studies specifically designed to validate our models' predictive ability in an even broader range of entomological and ecological settings would now be worth performing to strengthen the proposed approach, and to allow for its use in a large scale operational/policy setting.

## Supporting Information

Appendix S1 Method to estimate the probability of transmission when human incidence needs to be inferred from prevalence data.
(PDF)

Appendix S2 Implication of assuming constant entomological condition across houses.
(PDF)
