# Supplementary material for: The Improbable Transmission of Trypanosoma cruzi to Human: The Missing Link in the Dynamics and Control of Chagas Disease
Source: PLoS Negl Trop Dis. 2013 Nov 7;7(11):e2505. doi: 10.1371/journal.pntd.0002505 (PMC3820721; doi:10.1371/journal.pntd.0002505)
Supplement: Appendix S1 — Method to estimate the probability of transmission when human incidence needs to be inferred from prevalence data. (PDF) [file pntd.0002505.s001.pdf]

Appendix S1: method to estimate the probability of transmission T when human incidence needs to be inferred from prevalence data.

Because measuring directly the incidence (i.e. the number of new cases) requires a follow-up survey of the population, a problematic and yet common situation occurs where only humans' prevalence is known rather than a number of new cases. To circumvent the problem, one needs to solve the following question: what force of infection would lead to the observed prevalence.

By assuming a constant daily human mortality among ages, μ, one can approximate that the distribution $\varphi(a)$ of individuals aged a among the population is: $\varphi(a) = \mu e^{-\mu a}$. Also using the catalytic model again, in a cohort the proportion $p(a)$ of individual that have acquired the parasite and are aged a follows: $p(a) = 1 - e^{-\beta a}$. It follows that the prevalence P_h of infection among the total population is:

$$P_h = \frac{\int \varphi(a)\, p(a)\, da}{\int \varphi(a)\, da} = \frac{\beta}{\mu + \beta}. \qquad \text{Eq. A1}$$

By rearranging this formulae, we find the expression of the force of infection appearing in the main text, e.g. $\beta = \mu P_h / (1 - P_h)$, that can be used to estimate the force of infection for a given prevalence of infection measured in the whole human population.

From Eq. A1, we also conclude that the prevalence $P_{h,a<A}$ of infection among individuals aged less than A is:

$$P_{h,a<A} = \frac{\int \varphi(a)\, p(a)\, da}{\int \varphi(a)\, da} = \frac{\beta}{\mu + \beta} - \frac{\mu e^{-\mu A}\left(1 - e^{-\beta A}\right)}{(\mu + \beta)\left(1 - e^{-\mu A}\right)}. \qquad \text{Eq. A2}$$

This equation can be used to estimate numerically the force infection (β) when the prevalence of infection is measured only for a subset of the population (typically children). Combined with an estimate of the number of potentially infectious bites per individual, C, this allows estimating the probability of transmission using equation 8 of the main text.
