# Supplementary material for: The Improbable Transmission of Trypanosoma cruzi to Human: The Missing Link in the Dynamics and Control of Chagas Disease
Source: PLoS Negl Trop Dis. 2013 Nov 7;7(11):e2505. doi: 10.1371/journal.pntd.0002505 (PMC3820721; doi:10.1371/journal.pntd.0002505)
Supplement: Appendix S2 — Implication of assuming constant entomological condition across houses. (PDF) [file pntd.0002505.s002.pdf]

Appendix S2: implication of assuming constant entomological condition across houses

When estimating the probability of transmission from aggregated data (Cases 2 and 3) we must assume that the number of potentially infectious contacts with infected vector per human is the same in each house. In this section we discuss the implications of such an assumption on our estimate of the probability of transmission.

Starting from equation 1 in the main text and using the same notations, at the level of n households, we have:

$$\sum_n X_{j,t} \sim B\left(\sum_n S_{j,t}, r_t\right). \qquad \text{Eq. S2.1}$$

Thus taking the expectation:

$$E\left[\sum_n X_{j,t}\right] = \sum_n S_{j,t}\, r_t\,. \qquad \text{Eq. S2.2}$$

Assuming $TC_{j,t} \ll 1$ (verified in Rabinovich et al. 1990), Eq. S2.2 becomes:

$$E\left[\sum_n X_{j,t}\right] = T \sum_n \frac{S_{j,t} Y_j}{N_{h,j}} \qquad \text{Eq. S2.3}$$

with $Y_j = N_{v,j}\, P_{v,j}\, b_j\, F_j\, t$. By replacing $\frac{S_{j,t}}{N_{h,j}}$ with $\left(1 - P_{h,j}\right)$ where $P_{h,j}$ is the prevalence of infection among humans in house j, we write:

$$E\left[\sum_n X_{j,t}\right] = T \sum_n \left(1 - P_{h,j}\right) Y_j \qquad \text{Eq. S2.4}$$

which can also be written:

$$E\left[\sum_n X_{j,t}\right] = T\left[\left(1 - E\left[P_{h,j}\right]\right) \sum_n Y_j + n \operatorname{cov}\left(\left(1 - P_{h,j}\right), Y_j\right)\right]. \qquad \text{Eq. S2.5}$$

By assuming the covariance component (noted ‘cov’ above) is negligible compared to $\left(1 - E\left[P_{h,j}\right]\right) \sum_n Y_j$, we obtain:

$$E\left[\sum_n X_{j,t}\right] = T\left(1 - E\left[P_{h,j}\right]\right) \sum_n Y_j. \qquad \text{Eq. S2.6}$$

Equation Eq. S2.5 is equivalent to equation 5 in the main text assuming $TC_{j,t} \ll 1$.

While a full description of the error induced by the assumption based on further modelling is outside the scope of this paper; given that the dataset 1 (from Rabinovich et al. 1990) included the spatial information, we were able to estimate the bias.

For the assumption to hold, we must verify that in Eq. S2.5, $n \operatorname{cov}\left(\left(1 - P_{h,j}\right), Y_j\right)$ is negligible compared to $\left(1 - E\left[P_{h,j}\right]\right) \sum_n Y_j$. Using dataset 1, we estimated the force of infection β_j's for each house (as $\beta = \mu P_h / \left(1 - P_h\right)$) to obtain estimation of the $P_{h,j}$'s. Using these values we were able to estimate not only $E\left[P_{h,j}\right]$ and $\sum_n Y_j$, but also the covariance between household prevalence and the total number of potentially infectious contact per household (Y_j), the latter holding the information about households' heterogeneities. We could then formally compare $\left(1 - E\left[P_{h,j}\right]\right) \sum_n Y_j$ and $n \operatorname{cov}\left(\left(1 - P_{h,j}\right), Y_j\right)$ and found that:

$$\left(1 - E\left[P_{h,j}\right]\right) \sum_n Y_j \sim 5\, n \operatorname{cov}\left(\left(1 - P_{h,j}\right), Y_j\right).$$

Estimating T without accounting for the spatial correlation (e.g. with aggregated data) thus result in an underestimation of T by a factor 0.8. In other words, we have $T_{aggregated} \approx 0.8\,T$.

Given the small sample size and somewhat atypical situation described in Rabinovich et al. (1990), results should be interpreted with caution. We stress however that the situation in Rabinovich et al. (1990) is likely to be an extreme situation with high density of vector, high

prevalence and highly domiciliated vectors. The impact of neglecting the covariance is likely to be lower when density of vectors or prevalence are lower and when vectors are not domiciliated (creating a more uniform distribution of vector abundance across houses).
